# The CGRP-nociceptor axis in cancer: tumor-specific programs of immune evasion, stromal remodeling, and tumor growth

**DOI:** 10.3389/fonc.2026.1968354

**Published:** 2026-09-22

**Authors:** Yunqi Hua, Yubo Liu, Baochun Wang, Yanhong Liu, Xiaoling Tian, Fengqi Liu, Qinggang Tian, Fangrui Yin

**Affiliations:** 1 Department of Oncology, Baotou Cancer Hospital, Baotou, China; 2 Inner Mongolia Key Laboratory of Life Health and Bioinformatics, School of Life Science and Technology, Inner Mongolia University of Science and Technology, Baotou, China; 3 Baotou Medical College, Inner Mongolia University of Science and Technology, Baotou, China; 4 Department of Pharmacy, Binzhou Polytechnic University, Binzhou, Shandong, China; 5 Department of Rheumatology, The First Affiliated Hospital of Baotou Medical College, Baotou, China

**Keywords:** cancer neuroscience, cancer-associated fibroblast, CGRP, immunotherapy, neuroimmune signaling, nociceptor, tertiary lymphoid structure, tumor microenvironment

## Abstract

Cancer neuroscience has established that peripheral nerves can shape tumor growth and antitumor immunity, although the underlying mechanisms vary across malignancies. Calcitonin gene-related peptide (CGRP), released mainly by peptidergic nociceptors and sensed through the CALCRL/RAMP1 receptor complex, is emerging as a recurrent mediator of tumor-nerve communication. This review synthesizes current evidence for CGRP signaling across tumor types. In melanoma and head and neck squamous cell carcinoma (HNSCC), CGRP can directly suppress tumor-infiltrating T cell activity. In lung adenocarcinoma, it acts through a macrophage-fibroblast relay that limits tertiary lymphoid structure assembly. In medullary thyroid cancer, tumor-associated CGRP is linked to abnormal dendritic cell development. In pancreatic ductal adenocarcinoma, nociceptor-derived CGRP participates in a reciprocal interaction with cancer-associated fibroblasts that lowers IL-15 and suppresses natural killer cells. Additional studies identify tumor-intrinsic outputs, including gastric tumor growth, CGRP-dependent cytoprotective autophagy in oral cancer, and RAMP1-dependent growth in gastrointestinal cancer models. Liver data currently support a role in fibrogenesis, while the relevance of this pathway to hepatocellular carcinoma remains unresolved. Together, these findings support a shared nociceptor-CGRP signaling system with tumor-specific cellular outputs. CGRP-pathway drugs and nociceptor-directed interventions therefore warrant tumor-specific translational studies, although anticancer efficacy remains predominantly preclinical.

## 1 Introduction

Immune checkpoint blockade has transformed oncology, yet primary and acquired resistance remain common and motivate the search for regulatory systems beyond PD-1/PD-L1 and CTLA-4 (1, 2). Cancer neuroscience provides one such framework. Tumors are innervated structures in which neuronal activity, glial programs, vascular regulation, stromal remodeling, pain pathways, and immunity can interact bidirectionally (3–6). These interactions are not merely consequences of tumor growth: in selected models, perturbing nerves or neuronal signals changes tumor progression and antitumor immunity.

CGRP is a 37-amino-acid neuropeptide with established roles in vasodilation, nociception, and neurogenic inflammation. Its canonical receptor contains calcitonin receptor-like receptor (CALCRL) and receptor activity-modifying protein 1 (RAMP1), with an accessory receptor component protein supporting signal transduction (7). CGRP commonly couples to Gs-cAMP signaling, but the biological consequence depends on the responding cell and tissue context. In cancer, RAMP1 has been detected in immune, stromal, and malignant-cell compartments, creating multiple possible routes by which the same ligand can produce different outcomes.

Across tumor types, CGRP signaling engages distinct cellular targets and produces context-dependent biological effects. The current literature supports a shared upstream nociceptor-CGRP communication system whose downstream consequences are determined by the responding cell population and tissue context. Accordingly, this review integrates tumor-specific mechanisms while considering evidence from preclinical tumor models, human tumor analyses, migraine therapeutics, and cancer pain studies according to their respective experimental and clinical settings.

Unlike broad cancer-neuroscience and neuroimmune reviews, this review is organized around the identity of the CGRP-responsive cell, the tumor-specific circuit, and the strength of the supporting evidence. This framework separates shared signaling components from context-dependent outputs and emphasizes unresolved questions, including the specificity of broad neural interventions, the limitations of receptor-expression data, the restricted evidence for reversal of immune checkpoint blockade resistance, and the absence of demonstrated clinical anticancer efficacy.

To support this focused narrative review, PubMed/MEDLINE was searched from inception through 2 September 2026 using combinations of “calcitonin gene-related peptide,” “CGRP,” “RAMP1,” “CALCRL,” “nociceptor,” and “sensory neuron” with cancer-, tumor-, immune-, and tumor-type-specific terms. Reference lists of eligible primary studies and relevant reviews were also screened. Priority was given to peer-reviewed original studies that directly examined CGRP/CALCRL/RAMP1 or nociceptor interventions in cancer models, human tumors, single-cell or spatial datasets, and functional cell or organoid assays. Reviews and non-cancer studies were used only for receptor biology, physiological context, pharmacological tractability, and safety. Because the study designs were heterogeneous and no formal risk-of-bias synthesis was performed, this is reported as a focused narrative search rather than a systematic review. Targeted supplementary searches and checks of thematic article collections were completed on 11 September 2026 to contextualize immune regulation, biomarker development, therapeutic delivery, and normal tissue responses.

Cross-tumor comparison is therefore appropriate only at the level of pathway architecture: a CGRP signal reaches a receptor-competent cell and changes a local tumor program. The circuits are not directly interchangeable because the responding compartment differs among CD8 T cells in melanoma and HNSCC, macrophages in lung adenocarcinoma, dendritic cells in medullary thyroid cancer, CAFs in PDAC, and malignant cells in gastrointestinal and oral cancer models. Consequently, a result in one tumor type should not be extrapolated to another without confirming the ligand source, CALCRL/RAMP1 receptor competence, downstream response, and relevant disease stage.

## 2 Biological architecture of the CGRP-nociceptor axis

Peripheral sensory nerves can enter or expand within tumors, but the initiating signals are not identical across cancer types (5, 6, 8). In melanoma, cancer cells promote nociceptor neurite outgrowth, sensitization, and neuropeptide release (9). Gastric cancer models show NGF-dependent expansion of nociceptive nerves and functional connectivity between gastric tumor cells and CGRP-positive sensory neurons (10). Under nutrient stress, oral carcinoma cells use reactive oxygen species and c-Jun to increase NGF, conditioning nociceptive nerves for CGRP production (11). In HNSCC, a distinct immune-pressure response induces tumor ATF4-dependent SLIT2, which activates tumor-innervating and tumor-draining-lymph-node nociceptive circuits (12). These studies support recurrent tumor-to-nerve communication, but they do not justify a single universal upstream ligand.

The reported source of CGRP also requires tumor-specific qualification. Nociceptor-derived CGRP is supported in melanoma, gastric cancer, nutrient-stressed oral carcinoma, lung adenocarcinoma, PDAC, and several HNSCC/OSCC models (9–16). Medullary thyroid cancer instead shows tumor-associated CGRP expression (17), whereas gastrointestinal tissues contain CGRP near tumor cells and within a subset of malignant cells, leaving a potentially mixed source (18). In studies based on whole-tumor staining or global Calca deletion, the producing compartment remains unresolved and should not be assigned exclusively to neurons.

After release, CGRP can reach target cells expressing the CALCRL/RAMP1 receptor complex. Cell identity is decisive. RAMP1-positive CD8 T cells are the critical target in melanoma (9); a CGRP-responsive macrophage population organizes the relevant downstream effect in lung adenocarcinoma (14); dendritic cell development is altered in medullary thyroid cancer (17); cancer-associated fibroblasts mediate the link to natural killer cells in pancreatic ductal adenocarcinoma (15); and malignant cells themselves are important targets in gastric, colorectal, and oral cancer models (10, 11, 18). These observations define a common receptor framework in which cancer type and target-cell identity determine the downstream pathway.

**Figure 1** summarizes this context-dependent architecture. Direct and reversible inhibition of antitumor immune cells can be conceptualized as a checkpoint-like regulatory function in preclinical settings; this description does not imply equivalence to clinically validated immune checkpoints. Across the broader literature, CGRP also regulates stromal and tumor-intrinsic programs; the term CGRP-centered neuroimmune hub therefore captures the range of cellular outputs described across tumor types. In this review, the term “checkpoint-like” is restricted to this preclinical functional analogy and is not used to imply receptor homology, clinical validation, or equivalence to PD-1/PD-L1 or CTLA-4.

**Figure 1:**
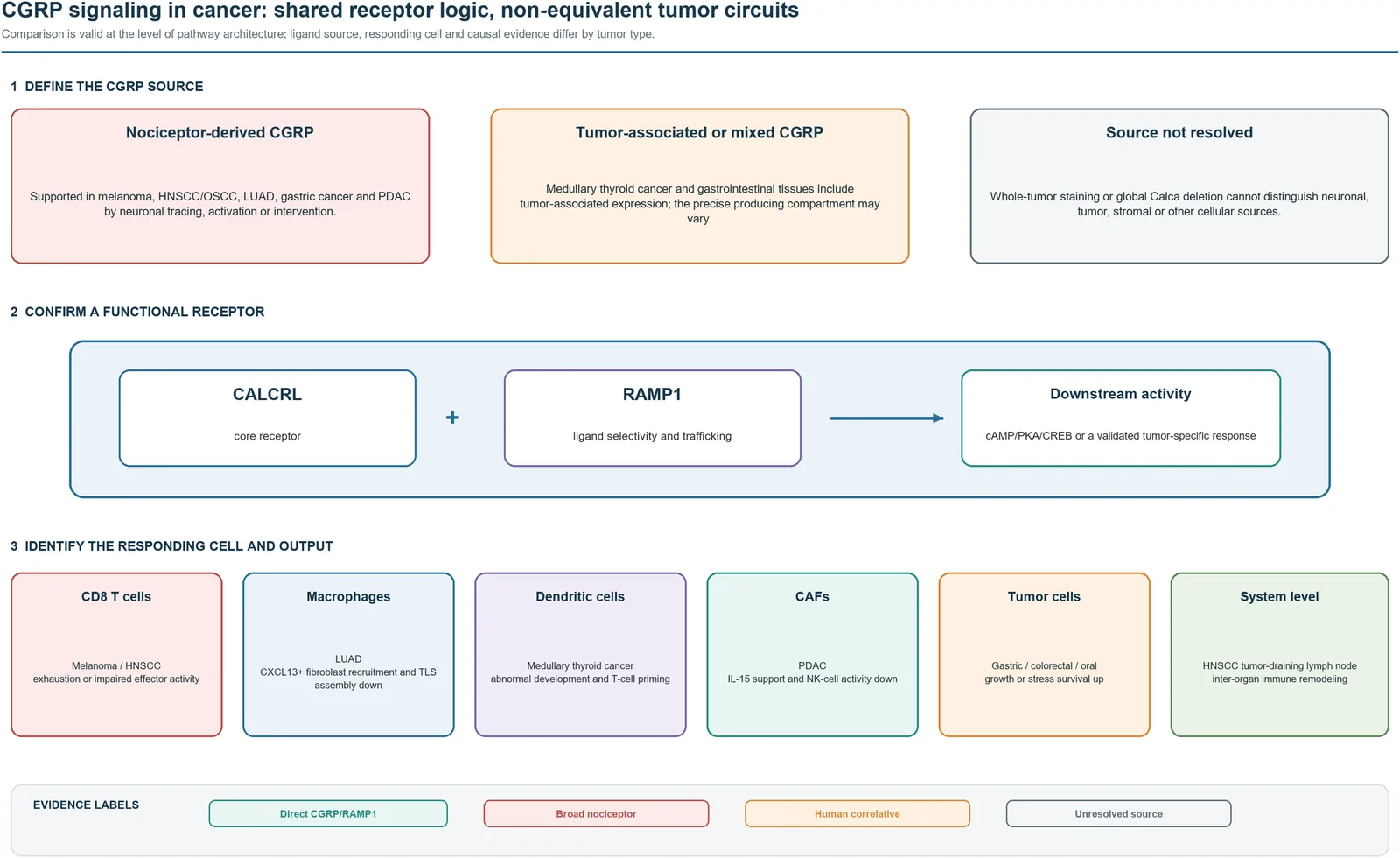
Shared receptor logic and tumor-specific boundaries of CGRP signaling in cancer. CGRP source is separated into nociceptor-derived, tumor-associated or mixed, and unresolved categories. Functional responsiveness requires CALCRL/RAMP1 receptor-complex formation and downstream activity rather than RAMP1 expression alone. Distinct responding cells generate non-equivalent outputs across melanoma, HNSCC/OSCC, lung adenocarcinoma, medullary thyroid cancer, PDAC, and gastrointestinal or oral cancer models. Evidence labels distinguish direct CGRP/RAMP1 manipulation, broad nociceptor intervention, human correlation, and unresolved source attribution.

The intervention used is critical for causal attribution. In this review, evidence is described as nociceptor-dependent when it is based on denervation, neuronal silencing, or lineage ablation without direct manipulation of the CGRP ligand or receptor. Evidence is considered CGRP-specific when convergent findings include ligand or receptor blockade, Calca or Ramp1 perturbation, or a functional rescue experiment. Because broad neural interventions alter multiple mediators, their effects are not attributed exclusively to CGRP unless CGRP-specific evidence is also available.

## 3 Tumor-microenvironment programs controlled by CGRP

### 3.1 Direct suppression of tumor-infiltrating T cells

The strongest direct neuropeptide-to-lymphocyte evidence comes from melanoma. Balood et al. showed that nociceptor-derived CGRP increases exhaustion of cytotoxic CD8 T cells. Genetic ablation of TRPV1-lineage neurons, local pharmacological silencing, and RAMP1 antagonism reduced immune exhaustion and tumor growth in B16F10 models. Ramp1-deficient CD8 T cells were protected in adoptive-transfer experiments, while single-cell analysis of human melanoma linked RAMP1-expressing intratumoral CD8 T cells to greater exhaustion and poorer prognosis (9). These data provide a causal chain from sensory neuron to CGRP to a defined immune-cell receptor and tumor outcome.

HNSCC offers convergent but independently generated evidence. Sensory nerve blockade by surgical, pharmacological, or genetic methods increased CD4 and CD8 T cell activity in orthotopic models, and CGRP directly inhibited CD8 T cell activity *in vitro (*16). In oral squamous cell carcinoma, global Calca deficiency reduced tumor growth and increased tumor-infiltrating CD4 T cells, CD8 T cells, and NK cells (19). Together, these studies indicate that CGRP regulates adaptive antitumor immunity in head and neck tumors through mechanisms that vary among experimental systems.

Recent OSCC evidence further links the sensory, pain, and immune components of this circuit. In a prospective observational cohort of 23 patients with tongue SCC, CGRP-positive nerve density and pretreatment pain were inversely associated with intratumoral CD8 T cell density; these human observations remain correlative. In syngeneic tongue-tumor models, persistent activation of peptidergic afferents promoted tumor growth and immune suppression, whereas local nociceptor ablation or systemic CGRP-receptor antagonism slowed tumor growth and improved antitumor immunity (13). At a broader level, OSCC combines tumor-secreted neuroactive mediators, trigeminal sensory activation, pain, perineural invasion, and immune remodeling. A recent review extends this framework to lncRNA-regulated neuroimmune interactions, but it should be interpreted as a conceptual synthesis rather than direct evidence for a CGRP-specific causal pathway (20).

### 3.2 Myeloid cells, dendritic cells, and lymphoid architecture

In lung adenocarcinoma, nociceptive innervation releases CGRP, which acts on a macrophage subset and impairs recruitment of CXCL13-positive fibroblasts. This prevents tertiary lymphoid structure (TLS) assembly, weakens B- and T cell-dependent immunity, and promotes tumor growth. Local sensory denervation restores TLS formation, and cigarette-smoke extract further activates the circuit. In smoke-exposed animals, pharmacological CGRP blockade sensitizes tumors to immunotherapy (14). These findings define a macrophage-to-fibroblast relay that links CGRP signaling to the organization of intratumoral immune architecture.

Medullary thyroid cancer illustrates a dendritic cell mechanism. Single-cell comparison with papillary thyroid cancer associated tumor CGRP expression with abnormal dendritic cell development, cAMP-related programs, increased KLF2, and impaired activity of tumor-infiltrating T cells. A CGRP-receptor antagonist offset the adverse effect on dendritic cell development *in vitro (*17). This supports a tumor-associated CGRP-DC axis, but not yet clinical antitumor efficacy.

A separate HNSCC study extends the field beyond the primary tumor. Under immune pressure, oral cancer cells activate an ATF4-SLIT2 program that stimulates tumor-innervating nociceptors and, in turn, nociceptors supplying tumor-draining lymph nodes. Increased lymph-node CGRP creates an immunosuppressed state, lowers CCL5, favors M2-like tumor-associated macrophages, and reduces immune checkpoint blockade efficacy. Targeting nociceptors or the ATF4-SLIT2-CGRP axis reverses these effects in preclinical models (12). This inter-organ pathway links tumor adaptation to lymph-node immune remodeling and represents a mechanism distinct from local CGRP-mediated T cell suppression.

### 3.3 A stromal relay suppressing natural killer cells in PDAC

In pancreatic ductal adenocarcinoma (PDAC), nociceptors and cancer-associated fibroblasts (CAFs) form a reciprocal CGRP-NGF interaction. This interaction reduces IL-15 expression in CAFs, decreasing NK cell infiltration and cytotoxic function and thereby promoting tumor progression and pain. In patient tissues, nociceptive innervation negatively correlates with NK cell infiltration and positively correlates with pain intensity, with prognostic associations for survival and relapse (15). Thus, in PDAC, CGRP regulates NK cell function principally through a stromal relay involving CAF-derived IL-15.

### 3.4 Tumor-intrinsic growth and stress-survival programs

Several studies show that the critical CGRP target is the malignant cell. In gastric cancer, NGF-dependent expansion of nociceptive nerves creates functional tumor-neuron connectivity; activating sensory neurons increases calcium signaling in cancer cells and promotes tumor growth and metastasis, whereas sensory-neuron ablation or CGRP inhibition suppresses tumor growth and extends survival in models (10). In nutrient-poor oral carcinomas, cancer-cell NGF conditions nociceptors to release CGRP, which induces cytoprotective autophagy through Rap1-mediated disruption of the mTOR-Raptor interaction. This circuit can blunt the effect of anti-glycolytic and anti-angiogenic nutrient-starvation therapies (11).

Human gastrointestinal evidence is also emerging. Analysis of 180 colorectal cancer, colorectal liver metastasis, and gastric cancer samples found RAMP1 in substantial fractions of tumor cells and linked RAMP1 expression to reduced survival. CGRP was detected near tumor cells and within some cancer cells, while CGRP stimulation increased growth of cell lines and patient-derived organoids in a RAMP1-dependent manner (18). Current evidence therefore supports a RAMP1-dependent tumor-growth program in colorectal and gastric cancer models, whereas the role of CGRP in inflammation-associated colorectal tumorigenesis remains unresolved. **Figure 2** compares six representative circuits in melanoma, HNSCC/OSCC, lung adenocarcinoma, gastric cancer, PDAC, and medullary thyroid cancer, with evidence-class labels.

**Figure 2:**
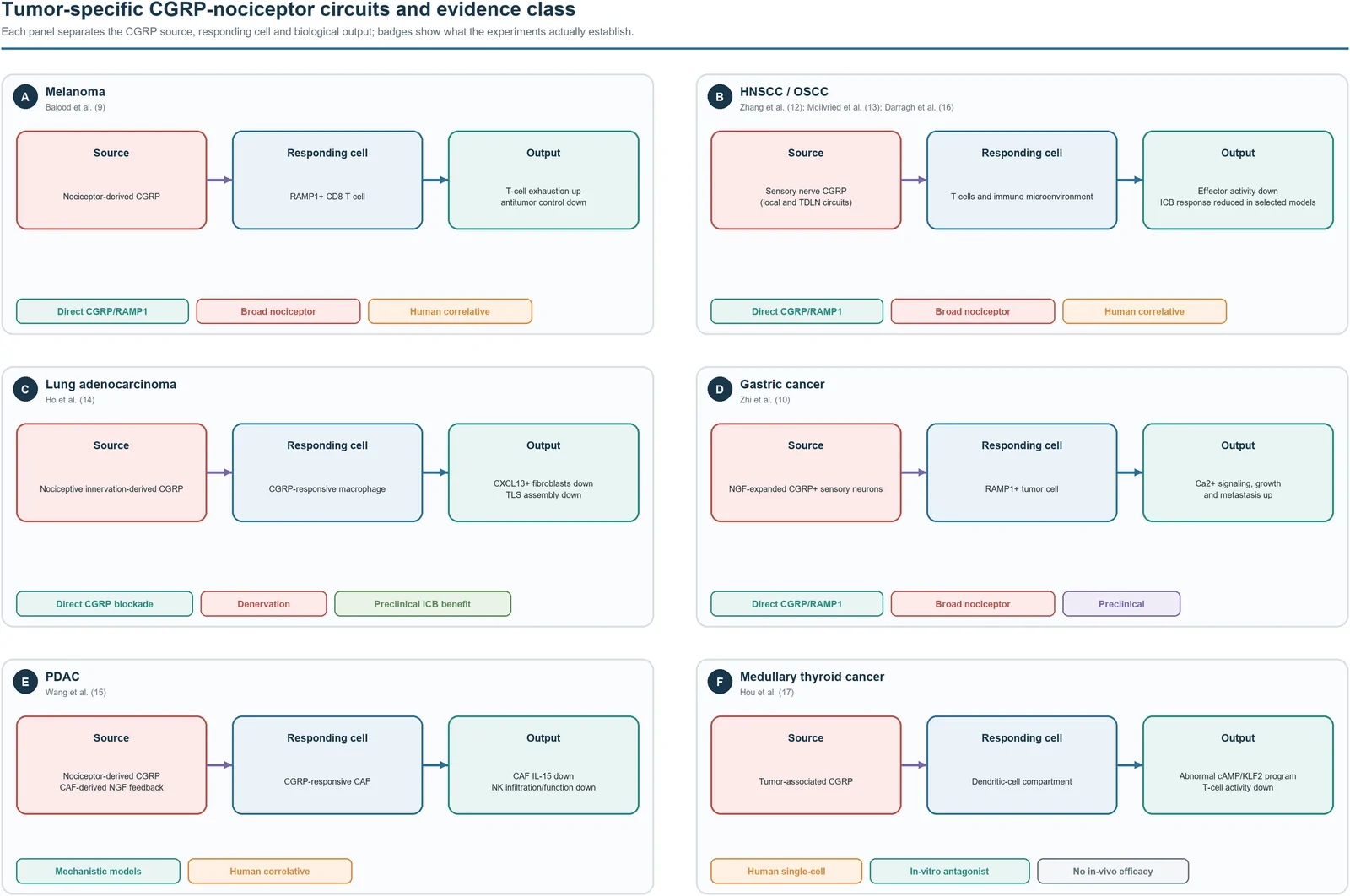
Tumor-specific CGRP-nociceptor circuits and evidence class. **(A)** Melanoma: nociceptor-derived CGRP acts on RAMP1-positive CD8 T cells (9). **(B)** HNSCC/OSCC: sensory CGRP contributes to local and tumor-draining-lymph-node immune suppression; direct CGRP-pathway and broader nociceptor evidence coexist with human correlations (12, 13, 16). **(C)** Lung adenocarcinoma: CGRP-responsive macrophages restrict CXCL13-positive fibroblast recruitment and TLS assembly, with preclinical ICB benefit after pathway targeting (14). **(D)** Gastric cancer: an NGF-dependent sensory circuit activates tumor-cell RAMP1 signaling (10). **(E)** PDAC: nociceptor-derived CGRP acts through CAFs to reduce IL-15 and NK cell support (15). **(F)** Medullary thyroid cancer: human single-cell findings and *in vitro* antagonist rescue support a tumor-associated CGRP-dendritic cell hypothesis without *in vivo* efficacy evidence (17).

## 4 Comparative evidence across tumor types

Across tumor types, a recurring communication structure is apparent: tumor- or stroma-derived signals activate nociceptors, CGRP is released locally, and a RAMP1-containing receptor on a context-relevant target cell alters tumor biology. The responding cell population and downstream program differ among malignancies. **Table 1** compares the cellular circuit, experimental context, and evidence interpretation for each tumor context.

**Table 1:** Evidence map for tumor-specific CGRP-nociceptor circuits.

Tumor context	Supported CGRP circuit	Evidence base	Evidence interpretation	Evidence class
Melanoma	Nociceptor CGRP -> RAMP1+ CD8 T cell exhaustion	Mouse models; human biopsy single-cell data	Causal preclinical evidence from neuronal and RAMP1-directed interventions; human single-cell findings are correlative.	Direct CGRP/RAMP1; broad nociceptor; human correlative
HNSCC/oral SCC	CGRP suppresses tumor-infiltrating T cells; a separate ATF4-SLIT2-CGRP circuit remodels tumor-draining lymph nodes	Human tumors plus syngeneic/orthotopic mouse models	Causal preclinical evidence for sensory nerve- or CGRP-dependent immune suppression; human tumor evidence remains mainly correlative.	Direct CGRP/RAMP1 in selected studies; broad nociceptor; global Calca; human correlative
Lung adenocarcinoma	CGRP-responsive macrophages -> impaired CXCL13+ fibroblast recruitment -> reduced TLS assembly	Mouse models; smoking-exposure model	Causal preclinical evidence from denervation and CGRP blockade; no clinical validation.	Direct CGRP blockade; denervation; preclinical ICB benefit
Medullary thyroid cancer	Tumor-associated CGRP -> abnormal DC development via cAMP/KLF2-linked programs	Human single-cell comparison; *in vitro* antagonist rescue	Human single-cell evidence is correlative, with functional *in vitro* antagonist rescue; no *in vivo* or clinical efficacy evidence.	Human single-cell; *in vitro* antagonist; no *in vivo* efficacy
PDAC	CAF NGF/nociceptor CGRP loop -> CAF IL-15 down -> NK infiltration and cytotoxicity down	Patient tissue correlations; mouse and cellular models	Mechanistic mouse and cellular evidence with human tissue correlations; the human associations are not causal.	Mechanistic mouse/cellular; direct pathway intervention; human correlative
Gastric cancer	NGF-dependent nociceptive expansion -> CGRP-RAMP1 signaling in tumor cells -> growth and metastasis	Multiple mouse models; 3D co-culture	Causal preclinical evidence from sensory neuron and CGRP-directed interventions; no clinical validation.	Direct CGRP inhibition; sensory ablation; preclinical
Colorectal/gastric cancers	CGRP and RAMP1 support growth of cancer cells and organoids	180 patient samples; cell lines; patient-derived organoids	Human prognostic associations plus functional cell-line and organoid evidence; clinical predictive value is unestablished.	Direct RAMP1 functional assays; organoid/cell-line; human correlative
Liver fibrosis	CGRP-RAMP1 -> HSC TGF-beta1/Smad2 and YAP -> fibrogenesis	Human diseased liver; mouse fibrosis model; HSC studies	Causal evidence in fibrosis models, not cancer models; relevance to HCC initiation or progression remains unresolved.	Fibrosis-only mechanistic evidence; not a cancer model

Evidence is categorized as causal preclinical when functional outcomes follow genetic, pharmacological, or denervation-based intervention; functional *in vitro* or ex vivo when activity is demonstrated outside an intact tumor model; and human correlative when based on tissue, prognostic, or single-cell associations. None of the listed circuits has established clinical anticancer efficacy.

Human tissue staining, single-cell RNA sequencing, spatial localization, and survival or pain associations can identify a compatible circuit but do not establish that CGRP causes tumor progression or treatment resistance. Causal attribution requires functional perturbation of the ligand or CALCRL/RAMP1 complex, demonstration of the predicted downstream response, and ideally lineage-restricted loss or rescue in an intact tumor model.

### 4.1 Immune-dominant contexts

Melanoma and HNSCC currently provide the clearest evidence that sensory signals can suppress adaptive antitumor immunity through CGRP (9, 12, 16, 19). Lung adenocarcinoma extends immune regulation to TLS organization (14), while medullary thyroid cancer links CGRP to abnormal dendritic cell development (17). These immune-dominant contexts justify studying CGRP as a preclinical checkpoint-like regulatory signal, but the target cell differs markedly across tumors. This terminology denotes an immunoregulatory analogy rather than equivalence to PD-1/PD-L1 or CTLA-4 blockade.

### 4.2 Stromal and tumor-intrinsic contexts

PDAC demonstrates stromal mediation: the relevant immune outcome is NK cell suppression caused by altered CAF support (15). Gastric cancer, oral carcinoma under nutrient stress, and colorectal/gastric cancer models emphasize tumor-cell growth or survival (10, 11, 18). These programs remain therapeutically relevant even though they do not fit a narrow immune-checkpoint definition. The broader term neuroimmune hub accommodates immune, stromal, and malignant-cell outputs without erasing their mechanistic differences.

### 4.3 Colorectal cancer and liver disease

For colorectal cancer, current direct evidence supports CGRP/RAMP1-associated tumor-cell growth in patient samples, cell lines, and organoids (18). The contribution of CGRP to colitis-associated colorectal tumorigenesis remains unresolved. In liver disease, CGRP-RAMP1 signaling promotes hepatic stellate-cell activation and fibrosis through TGF-beta1/Smad2 and YAP pathways in human diseased liver, mouse fibrosis, and cellular models (21). These findings establish a fibrogenic pathway with potential relevance to hepatocarcinogenesis, whereas its role in HCC initiation, immune regulation, tumor progression, and treatment response remains unknown.

### 4.4 Context dependence and neutral findings

The published cancer literature is enriched for tumor-promoting findings, so the scarcity of tumor-suppressive reports should not be interpreted as proof of a universal effect. Neutral results have been observed in immune-cold models: local RTX-mediated denervation did not significantly change growth of poorly immunogenic MOC2 OSCC, and systemic nociceptor ablation did not significantly alter poorly immunogenic YUMM1.7 melanoma in the reported experiments (9, 13). CGRP also has physiological immune-regulatory and tissue-protective functions outside cancer (7). Variation among studies may therefore reflect baseline immune competence, target-cell composition, ligand source, receptor functionality, tumor stage, dose and timing, and concurrent therapy. Each proposed circuit should be tested within its own tumor and treatment context.

## 5 Therapeutic strategies and their evidence level

### 5.1 CGRP ligand or receptor blockade

The migraine field has produced monoclonal antibodies against the CGRP ligand or receptor and small-molecule CGRP-receptor antagonists (gepants), demonstrating that the pathway is pharmacologically tractable in humans (22, 23). Erenumab targets the receptor complex, whereas fremanezumab and galcanezumab target the ligand. Rimegepant, ubrogepant, and atogepant are examples of oral receptor antagonists. In cancer models, genetic or pharmacological interference with CGRP-RAMP1 signaling reduces tumor growth or restores antitumor programs in melanoma, lung adenocarcinoma, gastric cancer, and oral cancer (9–11, 14). However, exposure, tissue penetration, dosing, and safety requirements in oncology may differ substantially from migraine prevention or treatment.

Long-term migraine experience, including 5-year open-label erenumab exposure, supports sustained pharmacological feasibility in a non-cancer population (24), but it does not establish anticancer efficacy or oncology-specific safety. Cancer studies must determine tumor and stromal penetration, effective intratumoral exposure, target engagement, dose intensity, treatment duration, interaction with standard therapy, and whether systemic blockade reaches the receptor-competent compartment identified for that tumor.

### 5.2 Nociceptor silencing or ablation

Upstream intervention can reduce multiple neuronal mediators at once. Local pharmacological silencing, sensory denervation, and genetic ablation of TRPV1-lineage neurons have shown antitumor or immune-restoring effects in preclinical models (9, 10, 14, 16). These approaches also carry a larger physiological footprint than ligand-receptor blockade because nociceptors participate in protective pain, tissue repair, vascular regulation, and host defense. Resiniferatoxin (RTX), an ultrapotent TRPV1 agonist, can functionally ablate a nociceptor subset. A first-in-human study supports intrathecal RTX as a potential opioid-sparing treatment for intractable cancer pain, with important adverse-event monitoring (25). Clinical RTX studies currently address analgesic efficacy, feasibility, and safety; tumor-control outcomes have not been evaluated.

### 5.3 Combination with immune checkpoint blockade

Combination therapy is most compelling when the CGRP circuit can be linked to a defined cause of ICB resistance. In smoke-exposed lung adenocarcinoma models, CGRP blockade restores TLS-related immunity and sensitizes tumors to immunotherapy (14). In HNSCC models, disrupting the ATF4-SLIT2-CGRP inter-organ circuit improves ICB responses (12). Melanoma provides a strong rationale by showing that RAMP1 signaling in CD8 T cells increases exhaustion (9). These findings provide a rationale for tumor-specific combination studies guided by evidence of an active CGRP circuit and a relevant resistance phenotype. Importantly, these findings represent three distinct evidentiary categories: CGRP-mediated immune suppression, association with an ICB-resistance phenotype, and experimentally demonstrated improvement of ICB response after pathway targeting. Direct improvement of ICB efficacy following CGRP or nociceptor targeting has been demonstrated only in selected lung adenocarcinoma and HNSCC models; the melanoma findings provide mechanistic rationale but do not by themselves establish combination efficacy. **Figure 3** summarizes the therapeutic strategies and the evidence level supporting each approach.

**Figure 3:**
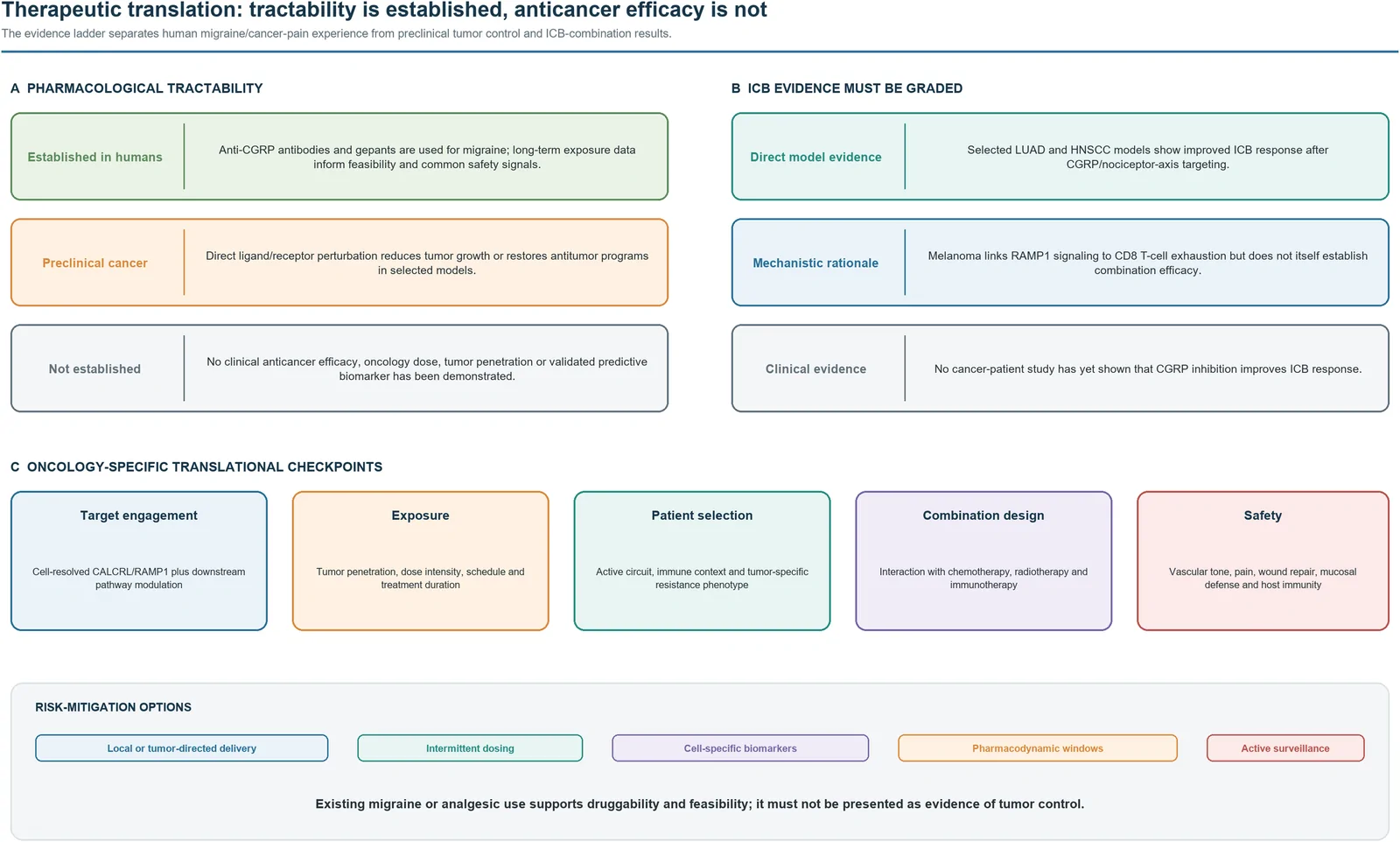
Therapeutic translation and evidence boundaries. Human migraine experience establishes pharmacological tractability of CGRP ligand/receptor blockade, and clinical RTX experience addresses cancer-pain analgesia; neither establishes tumor control. Direct improvement of ICB response after CGRP/nociceptor-axis targeting is limited to selected lung adenocarcinoma and HNSCC models, whereas melanoma provides mechanistic rationale. Oncology development requires tumor penetration, dosing, target-engagement, biomarker, combination, and safety studies, including surveillance of vascular homeostasis, protective pain, wound repair, mucosal defense, and host immunity.

## 6 Candidate biomarkers and patient selection

Biomarker development should identify the active circuit and the receptor-expressing cell population. RAMP1 expression can be evaluated in CD8 T cells in melanoma, macrophages in the LUAD TLS circuit, dendritic cells in medullary thyroid cancer, CAFs in PDAC, or malignant cells in gastrointestinal tumors (9, 10, 14, 15, 17, 18). Bulk-tumor RAMP1 measurements may obscure these cell-specific patterns. Spatial methods combining nerve markers, CGRP, RAMP1, and cell-identity markers may therefore provide greater mechanistic resolution than a single immunohistochemical score. RAMP1 expression alone is not a sufficient surrogate for functional CGRP signaling because receptor activity also requires CALCRL and appropriate downstream signaling competence. Where feasible, RAMP1 should therefore be interpreted together with CALCRL co-expression, pathway activation, and functional ligand- or receptor-perturbation data.

A practical validation hierarchy is: level 1, cell-resolved co-expression of RAMP1 and CALCRL; level 2, evidence of downstream signaling activity, such as cAMP/PKA/CREB engagement or a validated tumor-specific transcriptional response; and level 3, loss and rescue of the phenotype after ligand/receptor blockade, lineage-restricted deletion, or receptor re-expression. Only the third level provides strong functional causal validation. RAMP1-only RNA or protein measurements should remain hypothesis-generating and should not be labeled as proof of a functional CGRP receptor.

CGRP-positive nerve-fiber density is another candidate, particularly when paired with a mechanistic output such as CD8 exhaustion, TLS scarcity, low NK cell infiltration, or tumor-cell RAMP1 expression. TLS abundance may enrich for the lung-cancer circuit (14). Pain intensity can accompany active nociceptor signaling in PDAC and oral cancer (15, 26), although its limited specificity reduces its value as a standalone marker of CGRP-driven immune suppression. Circulating CGRP is attractive as a noninvasive marker, yet systemic concentration may not reflect local tumor release. None of these candidates is currently a validated predictive biomarker for anticancer CGRP therapy.

## 7 Challenges, controversies, and future directions

Three controversies cut across the available literature: whether effects of broad nociceptor interventions can be assigned specifically to CGRP, whether receptor-component expression identifies a functionally responsive cell, and whether CGRP-mediated immune suppression predicts reversal of ICB resistance. Resolving these questions requires tumor-specific causal experiments and human pharmacodynamic validation rather than extrapolation from a shared upstream signal alone.

Immune suppression is not specific to CGRP signaling. In colorectal cancer, macrophage-derived SPP1 suppresses CD8 T cell function through CD44-associated signaling (27). In hepatocellular carcinoma, ANXA2 supports CD63-dependent incorporation of PD-L1 into exosomes, which inhibit T cell activation (28). KRAS-mutant lung adenocarcinoma provides a tumor-intrinsic example in which ETV4 promotes PD-L1 expression (29). These mechanisms offer alternative explanations for immune dysfunction and poor treatment response. Studies of CGRP should therefore test its contribution alongside other local suppressive programs; shared immune endpoints alone cannot establish that these pathways belong to the same neural circuit.

### 7.1 Define the responding cell in each tumor

Future studies should combine lineage-restricted receptor perturbation, spatial profiling, and functional rescue to determine which cell actually receives CGRP. Whole-body Calca or Ramp1 deletion is informative but cannot by itself separate neuronal, immune, stromal, vascular, and tumor-cell effects. Reporting cancer type, disease stage, model, receptor-positive cell, intervention, and measured downstream output should become a minimum standard.

Global Calca deletion can alter peptide availability across anatomically distinct neuronal and non-neuronal sources, whereas global Ramp1 deletion affects immune, stromal, vascular, and malignant compartments and may change baseline physiology before tumor implantation. Conditional and inducible alleles, bone-marrow chimeras, adoptive-transfer designs, local pharmacology, and compartment-specific rescue experiments are needed to separate neuronal production from immune-, stromal-, vascular-, and tumor-cell reception.

Cell-resolved analyses can help distinguish these mechanisms. Integrated single-cell and spatial profiling has identified arginine-metabolism-associated mast-cell heterogeneity in breast cancer (30), while transcriptomic and single-cell analyses have linked GBP4-related programs to prognosis and cellular states in pancreatic adenocarcinoma (31). Single-cell profiling of colorectal cancer has likewise described diverse immune and stromal populations and inferred communication between them (32). Applied to CGRP research, such approaches could locate ligand sources and receptor-competent targets within competing microenvironmental programs. However, inferred ligand-receptor interactions and prognostic signatures require validation in the relevant cell compartment; functional results for another gene do not establish CGRP dependence.

### 7.2 Separate neural activation from nerve density

More nerve fibers do not necessarily mean greater CGRP release, and acute neural activity may be biologically important even without a density change. Studies should measure structural innervation together with neuronal activation, local CGRP release, receptor engagement, and the proposed cellular output. This is especially important when smoking, nutrient deprivation, treatment stress, or cancer pain may acutely activate sensory circuits (11, 12, 14, 26).

### 7.3 Build a translational evidence ladder

The studies reviewed here do not yet establish clinical anticancer efficacy for CGRP blockade or nociceptor ablation. Translational development should proceed through pharmacodynamic window studies, tumor-specific biomarker enrichment, and combination trials only where a defined resistance mechanism has been demonstrated. Migraine safety data can inform starting assumptions, while cancer pain RTX studies inform feasibility of selected neuronal interventions (22, 23, 25); neither substitutes for oncology dose finding, tumor-response endpoints, or long-term safety assessment.

Restoring immune recruitment offers a complementary therapeutic direction. Oncolytic viruses combine tumor-cell lysis with immune activation and can be engineered to deliver immunomodulatory signals (33). In syngeneic colorectal cancer models, a CCL19-expressing recombinant influenza virus increased immune infiltration, inhibited tumor growth and metastasis, and induced immune memory (34). These findings motivate testing whether local immune recruitment can complement removal of a demonstrated CGRP-mediated suppressive signal. Such combinations remain experimental hypotheses: the cited studies do not establish efficacy of combined oncolytic virotherapy and CGRP blockade, and neither therapeutic sequence nor the balance between antiviral and antitumor immunity can be assumed.

### 7.4 Protect physiological neuroimmune functions

CGRP and nociceptors participate in vascular homeostasis, mucosal defense, tissue repair, and protective sensation (7). Chronic pathway inhibition in patients receiving cytotoxic therapy or immunotherapy may have consequences not captured in migraine populations. Local delivery, intermittent dosing, receptor-cell selectivity, and tumor-restricted biomarkers may improve the therapeutic index.

Potential consequences of prolonged systemic inhibition include altered vascular adaptation and blood-pressure control, impaired protective pain and detection of tissue injury, delayed wound repair, reduced mucosal protection, and modified host defense. These risks may become more relevant during chemotherapy, radiotherapy, surgery, or immunotherapy, when tissue injury, infection risk, and inflammatory toxicities are already present. Oncology trials should therefore incorporate cardiovascular, neurologic, wound-healing, mucosal, infectious, and treatment-specific safety monitoring rather than relying solely on migraine safety experience.

Metabolic state and the identity of a delivered signal also matter when interpreting physiological effects. A neonatal metabolomics study associated early postnatal metabolic profiles with immune-cell subsets in preterm and term infants (35), illustrating context-dependent metabolic-immune associations rather than evidence about adult tumor immunity. In a separate regenerative setting, embryonic stem cell-derived exosomes delivered BDNF and promoted retinal Muller-cell retrodifferentiation through Wnt signaling (36). This provides a non-cancer example of a trophic signal acting through a defined cargo and recipient cell. Neither study evaluates CGRP inhibition; their relevance is limited to physiological context and the need to test immune and repair responses directly in oncology models.

## 8 Conclusion

The CGRP-nociceptor axis is a credible and therapeutically tractable component of cancer neuroscience. Current evidence supports a family of tumor-specific mechanisms linked by a shared upstream signal. The best-supported immune programs include CD8 T cell suppression in melanoma and HNSCC, TLS restriction through macrophage-fibroblast communication in lung adenocarcinoma, dendritic cell dysregulation in medullary thyroid cancer, and CAF-mediated NK cell suppression in PDAC. Gastric, colorectal, and oral cancer studies add tumor-intrinsic growth and stress-survival mechanisms, while liver evidence currently supports a role in fibrogenesis. This framework enables cross-tumor comparison while retaining cellular and mechanistic specificity. Clinical translation will require tumor-specific biomarker development, pharmacodynamic studies, dose-finding, and prospective evaluation of tumor-response endpoints. The pathway is pharmacologically tractable, but clinical anticancer activity has not been demonstrated; translation should proceed circuit by circuit rather than as a pan-cancer assumption.
